# Significant Improvement in Magnetorheological Performance by Controlling Micron Interspaces with High Permeability Submicron Particles

**DOI:** 10.1002/advs.202407765

**Published:** 2024-10-08

**Authors:** Tianxiang Du, Ning Ma, Zenghui Zhao, Yitong Liu, Xufeng Dong, Hao Huang

**Affiliations:** ^1^ School of Materials Science and Engineering Dalian University of Technology Dalian 116024 P. R. China; ^2^ School of Infrastructure Engineering Dalian University of Technology Dalian 116024 P. R. China

**Keywords:** bidisperse particles system, chain‐like structure, comprehensive performance, high permeability submicron particles, magnetorheological fluids

## Abstract

The shear yield strength, sedimentation stability and zero‐field viscosity of magnetorheological fluids (MRFs) are crucial for practical vibration damping applications, yet achieving a balanced combination of these performances remains challenging. Developing MRFs with excellent comprehensive performance is key to advancing smart vibration damping technologies further. Theoretically, incorporating a multiscale particle system and leveraging synergistic effects between their can somewhat enhance MRFs’ performance. However, this approach often faces issues such as insignificant increases in shear yield strength and excessive rise in zero‐field viscosity. In response, this study employs a DC arc plasma method to synthesize a high magnetic permeability, low coercivity submicron FeNi particles, and further develops a novel CIPs‐FeNi bidisperse MRFs. The introduction of submicron FeNi particles not only significantly enhances the shear2019 yield strength of MRFs under low magnetic fields but also promotes improvements in sedimentation stability and redispersibility without excessively increasing viscosity. Comprehensive performance analysis is conducted to explore the optimal content ratio, and detailed mechanisms for the enhancement of performance are elucidated through analysis of parameters such as chain‐like structure, magnetic flux density and friction coefficient. Most importantly, the superior comprehensive performance combined with straightforward fabrication methods significantly enhances the engineering applicability of the CIPs‐FeNi bidisperse MRFs.

## Graphical abstract

A unique bidisperse magnetorheological fluid (MRFs) is developed by blending self‐synthesized high magnetic permeability submicron FeNi particles with CIPs. Through the synergistic modification of particle properties and composition, this approach overcomes the challenges of achieving an optimal balance among key performance indicators such as dynamic yield strength (at low fields), sedimentation stability, and zero‐field viscosity, which are difficult to achieve with conventional MRFs. The study not only determined the optimal particle ratio but also detailed the mechanisms behind each critical performance improvement, thereby achieving a dual breakthrough in both high performance and simplified preparation.

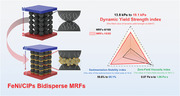

## 1 Introduction

In the realm of smart materials, magnetic fields responsive materials are widely regarded as the most promising due to their rapid response, cordless operation, significant changes and environmental friendliness.^[^ ^1^, ^2^, ^3^, ^4^, ^5^ ^]^ Among them, magnetorheological fluids (MRFs) are intelligent fluids whose rheological parameters such as viscosity and shear yield strength can be instantaneously and reversibly controlled by a magnetic field.^[^ ^6^, ^7^, ^8^ ^]^ The MRFs are typically composed of soft magnetic particles, carrier liquids and functional additives.^[^ ^9^, ^10^ ^]^ When no magnetic field is applied, the unmagnetized magnetic particles are uniformly distributed in the carrier liquid, resembling a Newtonian fluid. Upon the intervention of an external magnetic field, the magnetized magnetic particles align along the field lines, causing a rapid growth in the viscosity of the MRFs, akin to Bingham fluids. Upon removal of the magnetic field, owing to the soft magnetic properties of the particles and the rebound effect of the carrier liquid polymer chains, the MRFs will instantaneously return to its initial fluid state.^[^ ^11^, ^12^, ^13^ ^]^ The various properties of MRFs that change with the variation of magnetic field are referred to as the MR effect. Utilizing this effect, a plethora of intelligent devices can be developed for extensive applications in fields such as vehicles, buildings, bridges, military equipment, precision machine tools and beyond.^[^ ^14^, ^15^, ^16^ ^]^

The most typical application device of MRFs is the MR damper.^[^ ^17^, ^18^, ^19^ ^]^ By adjusting the shear yield strength of the MRFs through a magnetic field, control over the damping force output of the MR damper can be achieved. However, since the discovery of the MR effect by Rinbow in 1948, MR dampers have not achieved large‐scale engineering applications.^[^ ^20^, ^21^ ^]^ The fundamental reason lies in the difficulty of balancing key performance indicators of current MRFs, such as shear yield strength, zero‐field viscosity and sedimentation stability. Among them, shear yield strength determines the damping force that the MR damper can provide at different magnetic field strengths, zero‐field viscosity determines the injectability of the MRFs and the relative adjustment range of damping force, while sedimentation stability determines the long‐term performance stability of the MRFs. The element,^[^ ^22^ ^]^ morphology,^[^ ^23^ ^]^ particle size,^[^ ^24^ ^]^ content^[^ ^25^, ^26^ ^]^ and dispersibility^[^ ^27^, ^28^ ^]^ of soft magnetic particles are the main factors affecting the performance of MRFs. However, the influence of these factors on the key performance indicators is extremely complex, typically resulting in the improvement of one or two performance indicators accompanied by the deterioration of other performance indicators.^[^ ^29^, ^30^, ^31^ ^]^ Therefore, resolving the contradiction between key performance indicators of MRFs and preparing MRFs with high shear yield strength, satisfactory zero‐field viscosity and excellent sedimentation stability is an urgent bottleneck problem that needs to be addressed for the development of MR damping technology.

Particles are crucial for generating the MR effect, thus research on particles is the most extensive in the field of MRFs. Considering that the traditional use of microparticles inevitably results in numerous interspaces above submicron order between them, which are typically difficult to avoid by simply increasing the volume fraction of particles. The small size particles have smaller interspaces between them, but they lack the ability to form strong chain‐like structures suitable for MRFs applications under the influence of a magnetic field. Additionally, small‐sized particles based MRFs will lead to a sharp increase in zero‐field viscosity and deteriorated redispersibility.^[^ ^32^, ^33^ ^]^ Therefore, solely using either large or small‐sized particles cannot achieve MRFs with satisfactory comprehensive performance.

Preparing bidisperse particle system MRFs seems to offer a rational solution. In comparison to traditional MRFs, bidisperse MRFs involve introducing two different scales of magnetic particle components into the carrier liquids. Studies indicate that the bidisperse particle system significantly enhances the shear yield strength and sedimentation stability of MRFs.^[^ ^34^, ^35^, ^36^, ^37^ ^]^ This is attributed to the better filling of interspaces between large‐sized particles by subsistent small‐sized particles, resembling the strengthening principle of “interstitial solid solution”. Considering the cost, performance and practicality of MRFs, one choice for bidisperse particles system invariably includes commonly used micrometer‐sized carbonyl iron powders (CIPs), while the second option requires under submicron order particles to fill the interspaces. However, when employing nanoparticles to fill the interspaces, the zero‐field viscosity inevitably increases substantially due to their autoaggregation tendency, hindering improvements in the adjustability range and injectability performance of MRFs.^[^ ^32^, ^33^, ^38^ ^]^ Moreover, the expensive price of nanoparticles is detrimental to the actual need to reduce the cost of MRFs. Currently, small vehicles often require the damper internal coil to provide a magnetic field of ≈300 mT to overcome the issue of continuous work.^[^ ^39^ ^]^ This not only avoids the heating issue (lead to the performance depletion of the MRFs) caused by excessively high magnetic fields but also prevents the problem of excessive coil weight.^[^ ^40^, ^41^ ^]^ Consequently, the challenge posed to MRFs is to exhibit superior shear yield strength under slighter magnetic fields. Considering the satisfactory relative magnetic permeability of FeNi alloys at slighter magnetic fields, indicating that their significant magnetic flux density will rapidly achieve at slighter magnetic fields.^[^ ^42^, ^43^, ^44^ ^]^ Furthermore, fabricating FeNi into submicron order particles to fill the interspaces of CIPs, it seems promising to effectively address the issues of excessive viscosity and poor shear yield strength at slight fields present in current bidisperse MRFs.

In order to fully exploit the performance advantages of FeNi particles, this study employed the direct current (DC) arc physical method capable of producing high crystallinity, high purity particles, high‐yield, and achieved the preparation of narrow distribution submicron order particles through an airflow sieving process.^[^ ^42^, ^45^, ^46^, ^47^ ^]^ Subsequently, detailed characterization and analysis of the relevant properties of the initial particles, mixed particles and bidisperse MRFs were conducted. Furthermore, through comprehensive comparison of critical indicators, the optimal compounding proportion of CIPs to submicron FeNi particles was obtained. Ultimately, the reasons for the significant amelioration in the comprehensive performance (especially shear yield stress in slighter magnetic fields) of the MRFs were elucidated by combining the novel test results and the chain‐like structure model theory. This work undoubtedly provides important guidance for the design and preparation of excellent comprehensive performance MRFs.

## 2 Results and Discussions

### 2.1 Particles Crucial Characterization

**Figure 1:**
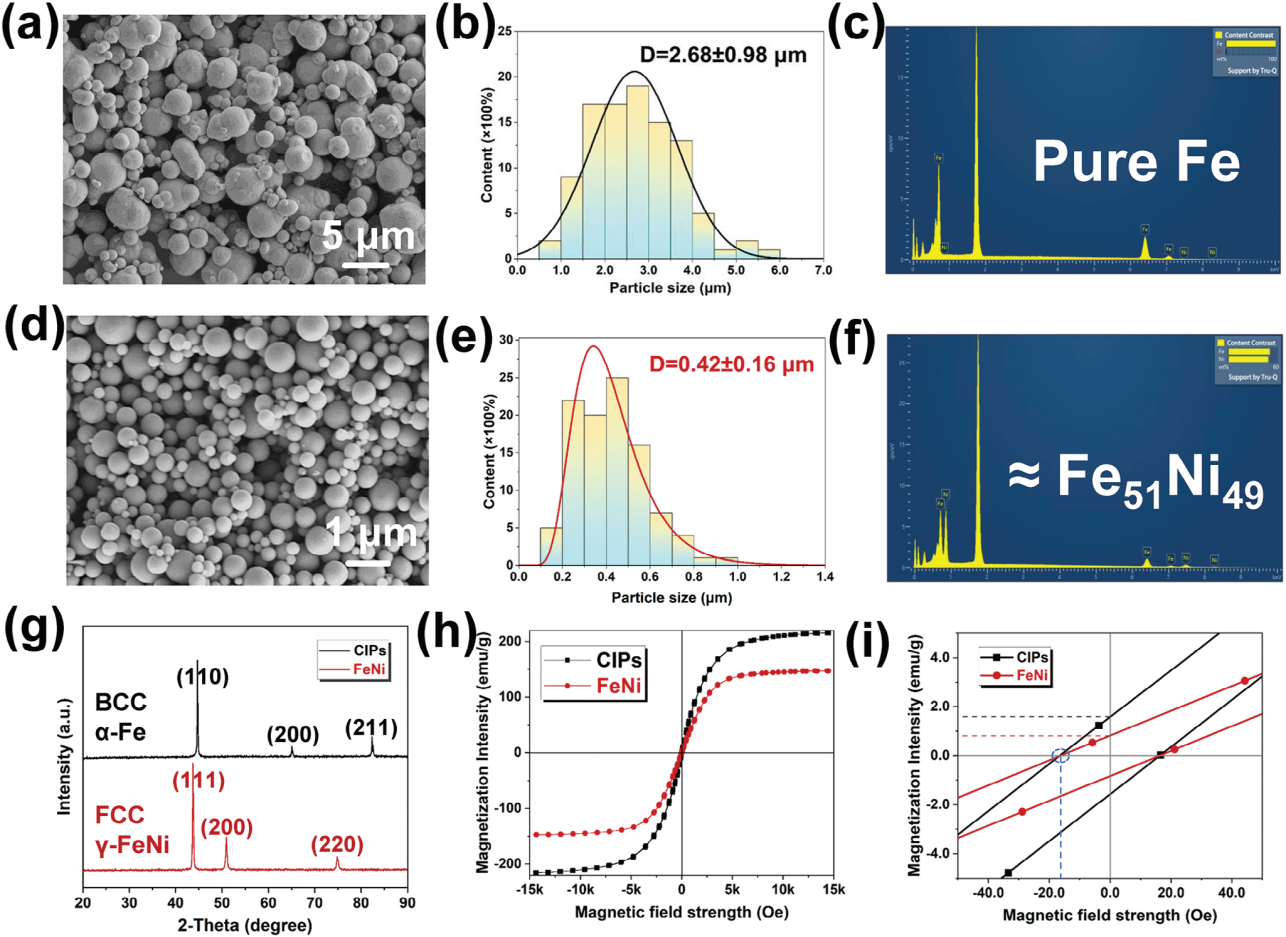
The basic performance characterization of the employed particles. The SEM, PSD and EDS images of CIPs a–c) and submicron FeNi particles d–f). The XRD, VSM and VSM of low magnetic field images of both particles g–i).

The CIPs and prepared FeNi particles were employed as the basic particles in this study, and their basic particle properties were characterized and compared. The microstructure, aggregation status and elemental distribution of CIPs and FeNi particles were comprehensively investigated using FE‐SEM and equipped EDS modules. The corresponding particle size distribution (PSD) was statistically analyzed using Image J software. **Figure** **1**a shows that the CN‐type CIPs exhibits a typical spherical structure with no apparent chemical bonding between particles, indicating an excellent dispersion. The PSD statistics (Figure 1b; Figure S1, Supporting Information) indicates that the average particle size of different batches CIPs is 2.68±0.98 µm, with an overall particle size range of 0.98 to 5.50 µm. The EDS analysis (Figure 1c) also confirmed that the CIPs consisted solely of Fe elements, with uniform distribution and no presence of other impurity elements. Similarly, the FeNi particles prepared DC arc method exhibit submicron‐scale dimensions, with an overall clear and uniform dispersion in spherical structures (Figure 1d). It can be seen from Figure 1e that the corresponding particle size ranges from 0.18 to 0.96 µm, with an average particle size of 0.42±0.16 µm. Moreover, through the statistics of PSD of different particles batches, it is found that there is no significant difference, which proves the batch stability of the prepared samples (Figures S2 and S3, Supporting Information). The EDS results of FeNi particles demonstrated that Fe and Ni elements were uniformly distributed within the particles, with a mass ratio of ≈1:1 (Figure 1f), indicating simultaneous precipitation of Fe and Ni elements in the particles prepared by this method.

Further investigation of the crystal structure and MR‐related magnetic properties was conducted through XRD and VSM. As shown in Figure 1g, XRD results reveal that the sharp peaks of CIPs correspond to JCPDS card No. 06–0696, with characteristic peaks located near 2*θ* = 44.78°, 65.21° and 82.60°, corresponding to crystal planes (110), (200) and (211), respectively. This phenomenon indicates that the α‐Fe phase was existed with a body‐centered cubic structure.^[^ ^46^, ^48^ ^]^ All sharp peaks of FeNi match with JCPDS card No. 47–1417, with diffraction angles 2*θ* values of 43.89, 50.87 and 74.65, which can be indexed to (111), (200) and (220) lattice planes. It is worth noting that when the Ni content in the FeNi alloy is greater than 10%, there will be an FCC transition from the BCC, and the 2*θ* value increases with the Ni content. In the same way, the sample explicitly exhibited that a face‐centered cubic (FCC) structure of γ‐FeNi phase was precipitation with high crystallinity and purity.^[^ ^49^, ^50^ ^]^

Figure 1h displays the VSM test curves of FeNi particles and CIPs, revealing that the saturation magnetization (*M*_s_) of CIPs can reach 215.3 emu g^−1^, whereas that of FeNi particles is 167.2 emu g^−1^. Figure 1i presents a comparison of the coercivity (*H*_c_) and remanence magnetization (*M*_r_) between FeNi particles and CIPs. Compared to the widely used CIPs in current engineering applications, the submicron FeNi particles exhibit a coercivity at the same level, with lower remanence magnetization, further indicating the applicability of the prepared particles. It is noteworthy that the *H*_c_ increases as the particle size decreases, but synthesized submicron FeNi particles still exhibit *H*_c_ values equal to CIPs (≈16.5 Oe), with *M*_r_ much lower than CIPs (0.8 vs 1.55 emu g^−1^). This phenomenon arises attributed to the higher magnetic permeability from the increased activity of Ni atom outer electrons in the FeNi alloy.^[^ ^43^ ^]^ The reason for choosing Fe_50_Ni_50_ in the study is to maximize the *M*_s_ of the particles while having a considerable magnetic permeability to ensure that the bidisperse particle system chain‐like structure forms a higher strength. At the same time, the prepared particles have significant advantages over other particles applied to MRFs. For details (refer to Table S1, Supporting Information). The above results signify the successful preparation of a bidisperse particle system meeting the envisioned requirements for the comprehensive performance of MRFs.

**Figure 2:**
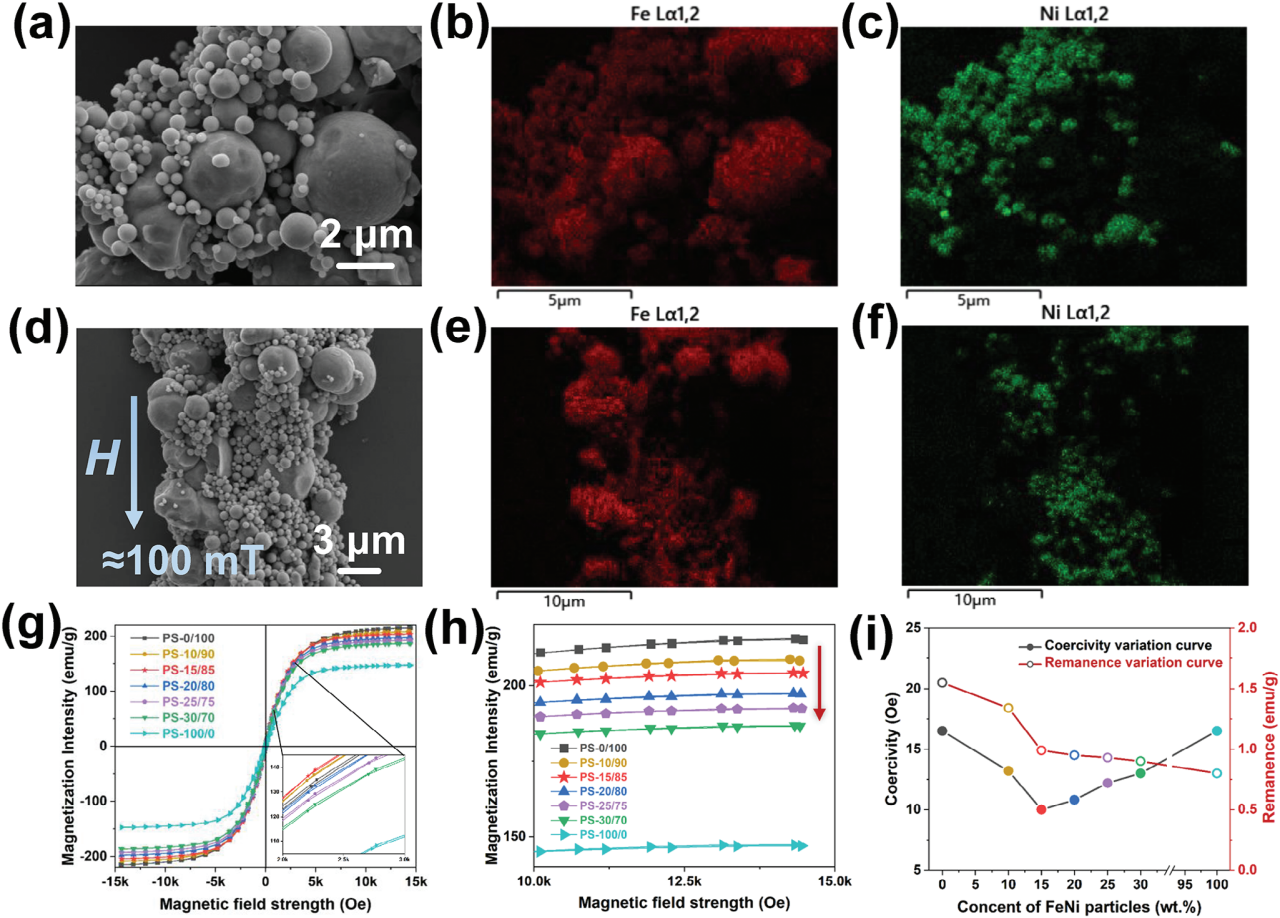
The performance characterization of the CIPs‐FeNi bidisperse particles systems. The SEM, EDS(Fe) and EDS(Ni) images of bidisperse particles systems without magnetic field a–c), the SEM, EDS(Fe) and EDS(Ni) images of bidisperse particles systems under magnetic field d–f), the VSM and saturation magnetization detail images of bidisperse particles systems g,h), the Coercivity and Remanence of bidisperse particles systems i).

To characterize the distribution state and magnetic properties after mixing two types of particles in different proportions, SEM, EDS and VSM‐related tests were conducted on the bidisperse particle system. Different particle systems are named according to the mass percentage as PS‐FeNi/CIPs, for example PS‐10/90. Based on the results of SEM and EDS shown in **Figure** **2**a, it can be observed that when no magnetic field is applied, the particles exhibit a relatively uniform distribution. The EDS of Figure 2b,c, also shows that submicron FeNi particles have weaker aggregation and distribution on CIPs surfaces and gaps. In order to study whether the particles can still be fairly evenly distributed under the action of magnetic field, we mixed two kinds of particles in a mass ratio of 1:1 with silicone oil, and then added them on the silicon chip, applying a magnetic field of 100 mT. After that, the silicone oil is quickly volatilized into a paste state by red light heating, so that a certain paste shape can be maintained after removing the magnetic field, and finally the SEM microscope is used for observation. When a magnetic field of ≈100 mT is applied (Figure 2d), the particles align along the direction of the magnetic field, forming a wide chain‐like structure dominated by CIPs, with submicron FeNi particles filling the remaining interspaces. In particular, submicron FeNi particles are mainly distributed in an aggregated‐like state in the interspaces of CIPs, only a small portion scattered on the surface, thereby increasing the volume utilization efficiency of the particle system. The distribution of elements after chain formation in Figure 2e and f also proves that the distribution between particles is consistent with what we have described.

Considering the magnetic properties of bidisperse particle systems are essential for better explaining the enhancement of MR effects and elucidating the reinforcement mechanism. From Figure 2g, it can be observed that prior to the 230 mT magnetic field, the magnetization intensity of the bidisperse particle system appeared slightly higher than that of pure CIPs. This phenomenon may be attributed to the increased volume utilization efficiency of the bidisperse particle system and the high magnetic permeability of FeNi particles under low magnetic fields. From Figure 2h, it can be seen that the *M*_s_ exhibits a decreasing trend with the introduction of submicron FeNi particles, attributed to the lower *M*_s_ value of the FeNi particles themselves. Differently, the *H*_c_ exhibited a trend of decreasing first and then increasing, as shown in Figure 2i. This phenomenon can be attributed to the increased volume utilization efficiency of the bidisperse particle system, which allowing the particles to accept an external magnetic field strength in a greater extent, thus facilitating the occurrence of demagnetization behavior. The *M*_r_ decreases with the increase of FeNi particles due to their high magnetic permeability and low *M*_r_ characteristics, ensuring the response speed and repeatability of the MRFs.^[^ ^51^ ^]^ In summary, the *H*_c_ values of the particles are all less than 20 Oe, and the *M*_r_ is less than 2 emu g^−1^, demonstrating that any compounding ratio studied is applicable to MRFs.^[^ ^52^ ^]^ Among them, PS‐15/85 exhibit the highest *M*_s_ (137.5 emu g^−1^ at 227 mT), the lowest *H*_c_ (10.1 Oe) and excellent *M*_r_ (0.99 emu g^−1^), which making it considered the most ideal ratio at present.

### 2.2 Crucial Performance Exposition of CIPs‐FeNi Bidisperse MRFs

**Figure 3:**
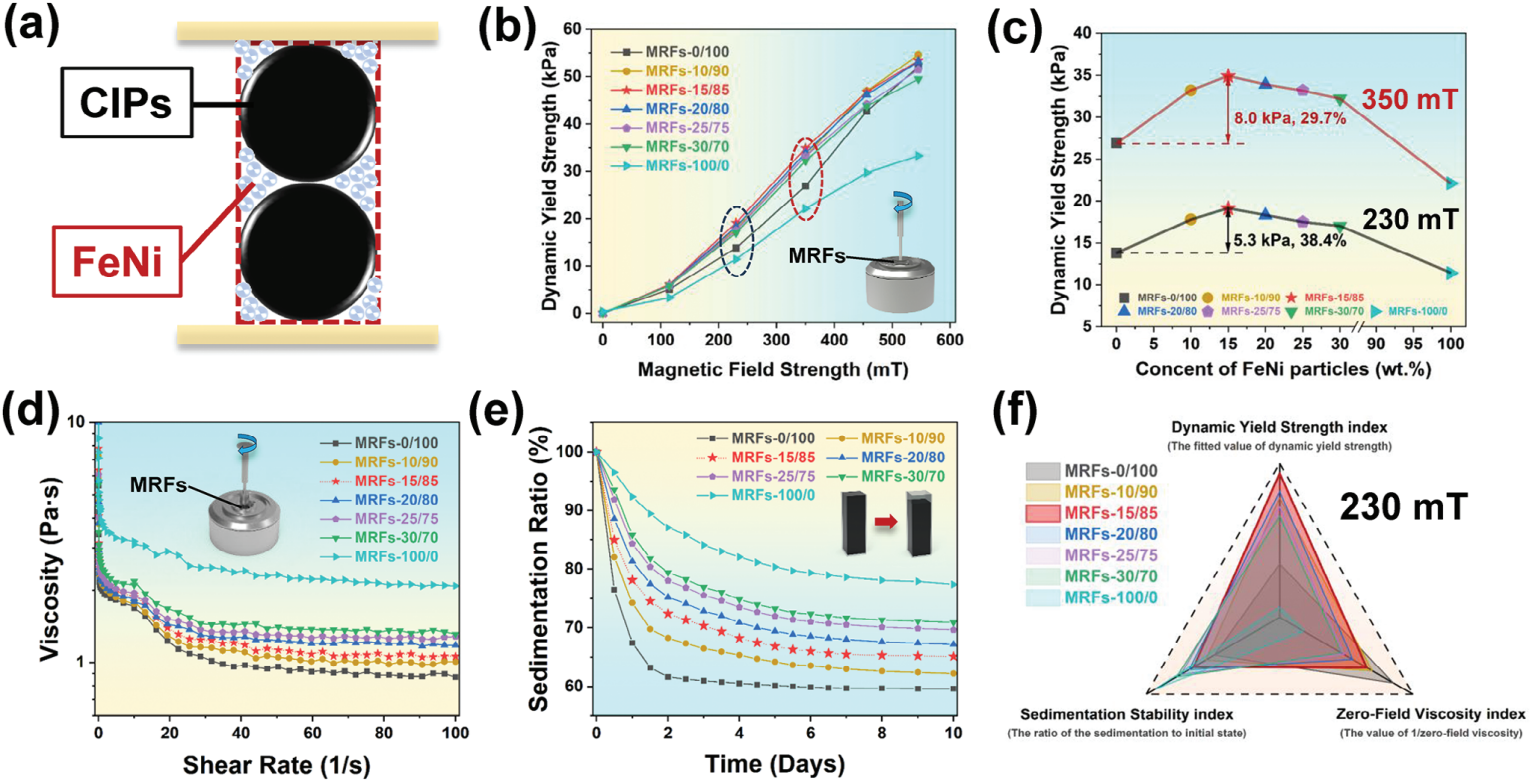
The key performance index values and comparison images of CIPs‐FeNi bidisperse MRFs. Theoretical calculation model of optimal proportion a), the Dynamic Yield Strength under different magnetic fields b), the Dynamic Yield Strength under 230 and 350 mT magnetic fields c), the Zero‐Field Viscosity test curves d), the Sedimentation Stability test curves e), the comprehensive performance comparison images of difference bidisperse MRFs f).

**Figure** **3**a describes the theoretical chain‐like structure state of CIPs and submicron FeNi particles under the action of a magnetic field, and conducts a preliminary analysis to calculate the optimal compounding ratio. Theoretically, the optimal state of particles chain is that large particles preferentially form the dominant chain‐like structure, and then small particles fill the generated chain‐like structure interspaces to form a more compact cylindrical‐like structure. At this point, the mass ratio should be evaluated based on the real density of the dominant particles (CIPs, 7.80 g cm^3^) and the tapped density of the filling particles (FeNi, 3.40 g cm^3^). Therefore, the theoretically optimal compounding ratio of CIPs: FeNi is ≈4:1. However, there may be deviations between actual results and theoretical values, attributed to factors such as particle size heterogeneity, irregular particle shapes, and reduced interparticle interactions in the presence of carrier fluid. Hence, further investigations will be conducted to explore the performance around the theoretical optimal compounding ratio.

The shear yield strength, zero‐field viscosity and sedimentation stability of MRFs are crucial performance indicators directly affecting the maximum damping force, adjustable range and long‐term performance stability of MR devices, respectively. Therefore, to determine the optimal balance of these indicators, a series of gradient ratios of CIPs‐FeNi bidisperse MRFs with a total particle mass percentage of 65% were prepared in this study. To simplify the research system, only dimethyl silicone oil was used as the carrier fluid without any additional additives. Particle systems were prepared according to the mass percentage of FeNi and CIPs, and the studied MRFs were named MRFs‐0/100, MRFs‐10/90, MRFs‐15/85, MRFs‐20/80, MRFs‐25/75, MRFs‐30/70 and MRFs‐100/0, respectively. Among them, MRFs‐0/100 was configured based on the specific particle composition of currently commercialized MRFs and served as the control group in this study.

The shear stress of CIPs‐FeNi bidisperse MRFs was tested using the parallel plate test system of MCR 301, and the shear yield strength values were obtained by extrapolating the Bingham model fitting curves,^[^ ^53^ ^]^ as shown in Figure 3b. As the FeNi particle content gradually increased, the shear yield strength did not exhibit a linear increase in various magnetic field ranges, demonstrating the non‐uniqueness of factors affecting shear yield strength. At low magnetic field intensity, the shear yield strength of MRFs increased with below 30% FeNi particles compared with that of single particle system. However, with the magnetic field intensity continues to increase, the MRFs‐0/100 will exhibit the highest shear yield strength. From Figure 3c, it can be seen that at moderate to low magnetic field strengths of 350 and 230 mT, the performance differences among MRFs with different compositions are most pronounced, with MRFs‐15/85 exhibiting the highest shear yield strength. At 230 mT, MRFs‐15/85 shows the highest increase ratio (38.4%) in shear yield strength compared to MRFs‐0/100, whereas at 350 mT it exhibits the highest absolute increase (8.0 kPa). This series of phenomena indicates that the high magnetic permeability characteristics of prepared FeNi particles and the interspaces filling effect of submicron particles are more advantageous for significantly enhancing the shear yield strength of bidisperse MRFs under moderate to low magnetic fields.^[^ ^54^, ^55^, ^56^ ^]^ However, with the continuous increase in magnetic field strength, the *M*_s_ of particles gradually becomes the primary factor affecting the shear yield strength, resulting in MRFs with higher CIPs content possessing higher shear yield strength. In the design of MR dampers, a quintessential objective is to achieve a satisfactory shear yield strength with the application of an internal magnetic field that is as minimal as possible. Such MRFs are more amenable to applications in miniature vehicles, as the magnitude of the magnetic field is predominantly determined by the number of turns in the coil. Utilizing MRFs with high yield stress at low magnetic fields can significantly reduce the generation of heat within the damper, thereby enhancing the controlled stability of the MRFs’ performance. Concurrently, the reduction in coil weight contributes to decreased fuel consumption and wear on the vehicle. Currently, the maximum magnetic field that dampers can typically apply is ≈300 mT, indicating that the MRFs‐15/85 prepared based on high magnetic permeability FeNi submicron particles will have significant advantages.

Figure 3d depicts the zero‐field viscosity variation curves of MRFs with different submicron FeNi particle contents, measured using the MCR 301 coaxial cylinder test system (CC27). The viscosity of MRFs of any particle composition reduces with incremental shear rate and then stabilizes gradually, exhibiting shear thinning characteristics.^[^ ^45^, ^57^ ^]^ In engineering applications, the viscosity value corresponding to a shear rate of 100 s^−1^ is typically used as the evaluation criterion for zero‐field viscosity. The corresponding zero‐field viscosity of the MRFs‐0/100 to MRFs‐100/0 series were 0.87, 0.98, 1.06, 1.18, 1.27, 1.32, and 2.09 Pa s, respectively. In the context of engineering applications, MRFs are often engineered to exhibit a reduced zero‐field viscosity to address the challenges associated with carrier fluid filling and to enhance the tunable range of the system. For miniature dampers designed to handle low loads, the structural design and sealing requirements necessitate a constrained size for the inlet hole. A smaller inlet hole size can impede the effective filling of the damper gap by viscous fluids, thereby compromising the optimal performance of the system. Additionally, to achieve a broader adjustable range, the design of the damper channel width is typically kept relatively narrow. This design choice underscores the importance of MRFs possessing superior fluidity to ensure optimal adjustment performance. However, when considering the comprehensive aspects of shear yield strength, MRFs‐15/85 are expected to demonstrate the most exceptional performance in practical applications.

**Figure 4:**
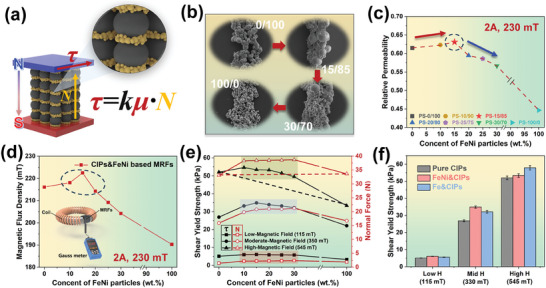
The mechanism explanation of significant increase in Shear Yield Strength for CIPs‐FeNi bidisperse MRFs under medium magnetic field. The Structure diagram and force analysis of bidisperse MRFs a), the SEM images of chain‐like structures at different particles proportions b), the relative permeability value (from VSM) at 230 mT parallel magnetic field c), the Magnetic Flux Density of different particles proportions MRFs in 230 mT magnetic field d), the relationship between Shear Yield Strength and Normal Force under different magnetic fields and particles proportions e), the Shear Yield Strength relationship of pure CIPs, CIPs‐FeNi and CIPs‐Fe MRFs at different magnetic fields f).

The sedimentation stability of CIPs‐FeNi bidisperse MRFs with varying submicron FeNi particle contents was evaluated, as shown in Figure 4e. To better reflect the real behavior of MRFs in non‐operational states, the measurement of sedimentation stability is reflected by the height of sediment deposited through natural settling (refer to Figure S4, Supporting Information). For a more precise comparison of the differences in sedimentation stability, the sedimentation ratio was recorded every 0.5 days over a total period of 10 days, and the pictures of the sedimentation condition for the corresponding days are given in Figure S5 (Supporting Information). Observationally, particles within MRFs initially exhibit rapid sedimentation, attributed to early‐stage particle agglomeration tendencies. As time progresses, the sedimentation rate of particles decelerates gradually, ultimately stabilizing. Moreover, higher concentrations of submicron particles correspond to slower sedimentation rates and improved sedimentation stability. This phenomenon arises due to enhanced Brownian motion of submicron particles and lower sedimentation driving forces. The ultimate sedimentation stability for MRFs‐0/100 to MRFs‐100/0 series were 59.6%, 62.2%, 65.1%, 67.2%, 69.6%, 70.9% and 77.4%, respectively. It is worth noting that compared with the commercial particle system MRFs‐0/100, MRFs‐15/85 exhibits a 38.4% increase in shear yield strength while still improving sedimentation stability by 9.3%. This indicates a significant advantage of such particle systems studied in balancing comprehensive performance. In the context of engineering applications, MRFs are typically engineered to possess superior sedimentation stability, which prevents the formation of rigid agglomerates of particles, thereby ensuring the stability and durability of the MRFs’ performance. This is particularly crucial for applications such as bridges and automotive suspensions that experience prolonged periods of stagnation. The utilization of bidisperse MRFs, which incorporate submicron particles with excellent soft magnetic properties, can more effectively mitigate performance degradation due to inter‐particle agglomeration and also prevent rapid overall sedimentation of particles.

To excel in engineering applications, MRFs typically need to simultaneously satisfy desirable dynamic yield strength, sedimentation stability and low zero‐field viscosity. Considering these factors comprehensively to select the optimal performance of MRFs, several reasonable indicators have been established to reflect the performance differences of MRFs, as shown in Figure 3f. To articulate the advantage about low viscosity, the reciprocal expression of zero‐field viscosity has been chosen as the corresponding index. Through rational analysis, it has been demonstrated that MRFs‐15/85 exhibits superior low‐field dynamic yield strength (19.1/34.9 kPa at 230/350 mT), enhanced sedimentation stability (65.1% at 10 days) and negligible increase in zero‐field viscosity (1.06 Pa s at 100 s^−1^). In light of the current extent achievable by pure CIPs MRFs (MRFs‐0/100), the utilization of the particle system MRFs‐15/85 will further surpass the current comprehensive performance of MRFs. Notably, this is compared to our previous work introducing FeCo nanoparticles,^[^ ^45^ ^]^ the bidisperse MRFs prepared in this study showed a more prominent yield strength (19.1 kpa) under a magnetic field suitable for miniature vehicle applications (≈300 mT), which was 30% higher than that of FeCo nanoparticles (14.2 kpa) under the same magnetic field. In addition, the selection of submicron FeNi particles solves the problem of excessive increase in nanoparticle viscosity, where the zero‐field viscosity is reduced from 1.25 to 1.06 Pa s, while reducing production costs. By investigating and comparing the properties of MRFs of other bidisperse particle systems, it is further proved that the comprehensive properties of MRFs prepared in this study have significant advantages (refer to Table S2, Supporting Information).

### 2.3 Enhancement Mechanism Analysis of CIPs‐FeNi Bidisperse MRFs

The above characterization demonstrates the satisfactory comprehensive performance of the prepared CIPs‐FeNi bidisperse MRFs, particularly in terms of shear yield strength at medium to low magnetic fields. Therefore, further elucidation will be given on the operational mechanism of bidisperse MRFs at medium to low magnetic fields, and explain the reasons for the optimal shear yield strength exhibited by MRFs‐15/85. Initially, particles form structures resembling chains/columns under the influence of a magnetic field, which possess a certain strength and constitute the fundamental reason for the shear yield strength of MRFs.^[^ ^58^ ^]^ Consequently, shear yield strength naturally depends on the normal stress (*N*) and the magnetically induced friction coefficient (*µ*) between particles forming chain‐like structures, and exhibiting a directly proportional relationship. As illustrated in **Figure** **4**a, upon introducing submicron particles in a certain amount, they tend to distribute within interspaces dominated by micron particle formed chain‐like structures under the action of corresponding magnetic field.^[^ ^59^, ^60^ ^]^ The filling of undesired interspaces will significantly improve both the friction coefficient between particles and the stress concentration phenomenon at chain‐like structure defects.^[^ ^61^ ^]^ Meanwhile, the overall density of the chain‐like structure will increase further, facilitating more interaction with the magnetic field and promoting the transformation of more chain‐like structures into column‐like structures. It is worth noting that this study utilized high magnetic permeability FeNi particles, which will further enhance the strength of the chain‐like structure compared to other soft magnetic particles.^[^ ^62^ ^]^ Theoretically, the introduction of submicron FeNi particles will enhance the shear yield strength of the MRFs from various aspects. As depicted in Figure 4b, the morphologies of chain‐like structures in MRFs doped with varying amounts of submicron FeNi particles were captured by SEM and EDS (refer to Figure S6, Supporting Information). Initially, with an increase in submicron FeNi particle content, the interspaces within the chain‐like structures formed by CIPs were effectively filled, resulting in a dense chain‐like structures. However, as submicron particles progressively replaced CIPs, the original CIPs‐dominated framework of chain‐like structures was gradually covered until it disappeared. Considering the performance of shear yield strength, the introduction of a small amount of submicron particles can synergistically enhance the strength of the chain‐like structures with CIPs. When the content of submicron particles exceeds a certain threshold, they will occupy vacant sites originally filled by CIPs in free state, making it difficult to form the initially CIPs based chain‐like structure and thereby preventing the formation of a robust main supporting framework. Meanwhile, the macroscopic and microscopic behaviors of MRFs‐15/85 under different magnetic fields were studied, as shown in Figure S7 (Supporting Information). Macroscopically, MRFs exhibit a decrease in fluidity with the increase of the magnetic field, and a parallel alignment structure to the direction of the magnetic field gradually emerges on the surface of the MRFs. Microscopically, with the increase of the magnetic field, the chain‐like structures of the MRFs particles tend to form thicker columnar structures, which is consistent with the observed macroscopic behavior of the MRFs. These test results contribute to a better understanding of the reasons for the increase in the yield strength of MRFs under the influence of a magnetic field.

To explore the significant distinctions in shear yield strength of CIPs‐FeNi bidisperse MRFs under medium to low magnetic fields, additional tests were conducted under some notably performance variable magnetic field. As shown in Figure 4c, relative magnetic permeability at 230 mT was computed from VSM curves for different particle compositions. The results indicate a trend of increasing and then decreasing relative permeability with the introduction of submicron FeNi particles, with PS‐15/85 exhibiting the highest relative permeability. Theoretically, relative permeability is primarily influenced by particle composition and size, where a decrease in particle size often leads to increased particle spacing, thereby causing a corresponding gradient decrease in relative permeability. The phenomenon of initial increase followed by decrease in relative permeability precisely illustrates that the change in particle packing density is conducive to enhancing relative permeability to a certain extent.^[^ ^63^ ^]^ However, this phenomenon was not observed under a high 545 mT magnetic field (refer to Figure S8, Supporting Information), indicating the advantage of FeNi particles only under low magnetic fields. It also demonstrates that under high magnetic field conditions, *M*_s_ and particle size have a more obvious influence on shear yield strength. Similarly, Figure 4d reflects the magnetic flux density changes of MRFs under a 230 mT parallel magnetic field. Similar to the VSM curves of particles, MRFs with 15 wt.% FeNi submicron content still exhibits the highest magnetic flux density, thereby excluding the influence of the carrier fluid. Unlike relative permeability, magnetic flux density shows a trend of initial increase followed by decrease under a high 545 mT parallel magnetic fields (refer to Figure S9, Supporting Information), indicating that the permeation capability of magnetic field is primarily influenced by particle chain‐like structure density. The bidisperse MRFs prepared in this study utilizes the advantage of FeNi particles reaching magnetic saturation quickly under tiny magnetic fields, which will enhance the output damping force of MR dampers under current low magnetic field applications. Therefore, the improvement of the particle system's permeability to magnetic fields can also to some extent overcome the disadvantage of low saturation magnetization of FeNi particles.

The normal stress refers to the magnitude of the force acting along the direction of the magnetic field on the rotor plane when measuring the MRFs, perpendicular to the direction of shear yield stress. Normal stress is primarily used to reflect the strength of the chain‐like structure distributed along the magnetic field direction and can also indicate the overall density of the chain‐like structure. To demonstrate the influence of factors affecting the yield strength of bidisperse MRFs, the relationship between shear yield strength and normal stress under different magnetic fields was tested, as shown in Figure 4e. It can be observed that both shear yield stress and normal stress increase with the magnetic field, indicating further enhancement of the magnetically induced chain‐like structure. Under low magnetic fields, the trends in shear yield stress and normal stress values are essentially consistent. As the magnetic field increases, there begins to be inconsistency between the changes in shear yield stress and normal stress. A comparison with MRFs containing extreme FeNi content at maximum magnetic field (5 A, 545 mT) reveals that while their normal stress values are similar, there is a significant discrepancy in shear yield strength. This suggests a notable difference in the friction coefficient between particles, which mainly depends on the magnetically induced interactions between particles and is directly proportional to them.^[^ ^61^ ^]^ Furthermore, as shown in Figure 4f, a comparison was made between MRFs composed of submicron Fe particles of the same size (refer to Figure S10, Supporting Information for VSM comparison, PSD and EDS analysis) in the same doping proportions series. It was found that under a moderate magnetic field of 350 mT, the shear yield strength of the MRFs containing mixed submicron FeNi particles was greater than that of the fluid containing mixed submicron Fe particles, while under a high magnetic field of 545 mT, the opposite trend was observed, though both were higher than that of the pure micron‐sized CIPs based MRFs. These phenomena can be attributed to two conclusions and explanations based on two underlying mechanisms. First, bidisperse particle systems enhance chain‐like structure strength, thereby increasing the shear yield strength of the MRFs. Second, the magnetic saturation that particles can achieve under the corresponding magnetic field determines the impact on shear yield strength. The increase in magnetization strength can be influenced by particle properties themselves or by the magnetic flux density under the identical magnetic field. Therefore, the discrepancy in magnetization intensity between FeNi particles and Fe particles under different magnetic fields enables the respective bidisperse MRFs to be applied in various fields. Additionally, an increase in overall density of the chain‐like structure will increase magnetic flux density, allowing the particle system to achieve higher magnetization strength under the identical magnetic field, thereby further increasing shear yield strength of the MRFs.

**Figure 5:**
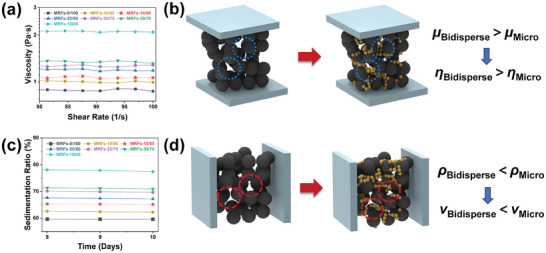
The mechanism explanation of Sedimentation Stability and Zero‐Field Viscosity change for CIPs‐FeNi bidisperse MRFs. The Zero‐Field Viscosity amplification curves and mechanism explanation model of MRFs a,b), the Sedimentation Stability amplification curves and mechanism explanation model of MRFs c,d).

The study elucidates the reasons behind changes in zero‐field viscosity and sedimentation stability of MRFs upon introducing bidisperse particles system. Regarding zero‐field viscosity, the introduction of submicron FiNi particles gradually increases the viscosity of MRFs (**Figure** **5**a). Due to their higher specific surface area, smaller submicron particles tend to self‐agglomerate or adsorb in the interspaces of micron‐sized particles, enhancing particle contact and increasing frictional resistance to particle movement (Figure 5b).^[^ ^45^, ^61^ ^]^ Simultaneously, the lower loose packing density of submicron particles allows the fluid to achieve a higher volume fraction under the same mass fraction. These behaviors collectively contribute to the increase in zero‐field viscosity. However, the introduced submicron FeNi particles, characterized by very low coercivity and excellent dispersion, mitigate extensive clustering between CIPs without significantly increasing the zero‐field viscosity of the particles themselves. To some extent, the bidisperse particles system of MRFs compensates for the drawbacks associated with the increase in zero‐field viscosity by enhancing its redispersibility capability.

As shown in Figure 5c, the sedimentation stability of MRFs gradually increases with an increase in the content of submicron FeNi particles. Particle sedimentation is a spontaneous process primarily driven by gravity minus resistance. According to Stokes’ law, effective prevention of sedimentation typically requires reducing the dead weight of particle or enhancing interactions between particles and the carrier liquid. As illustrated in Figure 5d, as submicron particles continuously fill the interspaces and surfaces of micron‐sized particles within the system, the overall density decreases accordingly. With increased Brownian motion due to smaller particle size, the overall suspension stability of particles is significantly improved.^[^ ^64^ ^]^ The changes of zero‐field viscosity and sedimentation stability exhibit the predictable opposite, which indicates that the equilibrium relationship must be considered in the selection of MRFs.

### 2.4 Other Performance Analysis of CIPs‐FeNi Bidisperse MRFs

In this study, MRFs‐15/85 is identified as possessing the optimal comprehensive performance, warranting further investigation into its application potential. Therefore, the flow curves, static yield strength and time responsiveness were measured under different magnetic fields, the reversible and power‐law function fittings were analyzed through a certain theory. To underscore the superiority of the bidisperse particle system, performance of MRFs‐0/100 was tested as a control (refer to Figure S11, Supporting Information).

**Figure 6:**
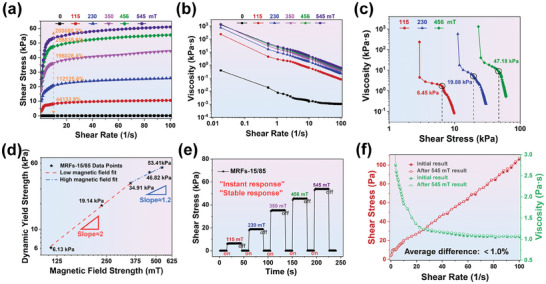
The other MR performance curves of MRFs‐15/85 at steady state mode. The curve of shear stress versus shear rate a), viscosity versus shear rate b), static yield strength c), dynamic yield strength d), time responsiveness images e) and reversibility f) of MRFs‐15/85.

In **Figure** **6**a and b, the variation curves of shear stress and viscosity of MRFs‐15/85 under different shear rates were investigated to facilitate optimal control under various operational conditions. In Figure 6a, shear stress exhibits a Newtonian fluid behavior with no yield stress and linear variation at zero magnetic field, transitioning to a Bingham fluid state with a distinct yield stress upon applying a magnetic field.^[^ ^65^, ^66^ ^]^ Corresponding to the magnetic field strengths of 115, 230, 350, 456, and 545 mT, the relative MR effects are 44 133.9%, 112 125.6%, 196 028.4%, 256 835.6% and 288 680.2%, respectively, which are significantly higher than those of MRFs‐0/100 (Figure S11a, Supporting Information) at low and medium magnetic fields. As shown in Figure 6b, both types of fluids demonstrate shear thinning behavior, where viscosity decreases with increasing shear rate.^[^ ^46^, ^57^ ^]^ For MRFs‐15/85, both shear stress and viscosity exhibit typical increases with magnetic field intensity. It is noteworthy that compared to MRFs‐0/100, MRFs‐15/85 shows higher magneto‐induced viscosity and shear stress values (refer to Figure S11a,b, Supporting Information). This can be attributed to the contribution of bidisperse particle systems to the strength of chain‐like structures.

Static yield strength is typically reflected from the connection between magneto‐viscosity and shear stress under CSS mode, with the relevant test results for MRFs‐15/85 shown in Figure 6c. The static yield strength is defined by the abrupt change in viscosity corresponding to the onset of structural breakdown in the initial chain‐like structures.^[^ ^30^, ^67^ ^]^ Therefore, the observed steady decrease in viscosity at low shear stresses is attributed to deformation of these chain‐like structures. For magnetic fields of 115, 230, and 456 mT, the static yield strengths of MRFs‐15/85 are 6.45, 19.88, and 47.18 kPa, respectively. Due to greater obstacles to be overcome for initial structural breakdown, the corresponding values of dynamic yield strength are all lower than the static yield strength. As shear forces acting on MRFs vary continuously in practical working mode, dynamic yield strength is often used to gauge the actual operational capability. Each type of MRFs has a critical magnetic field strength, denoted as *H*_critical_, which distinguishes the degree of variation in dynamic yield strength.^[^ ^45^, ^68^ ^]^ Below *H*_critical_, dynamic yield strength changes rapidly with the magnetic field, approximating a power law with an exponent close to 2. Beyond *H*_critical_, this rate slows down, typically with a power law exponent ≈1.5 for MRFs. The power‐law functions (1,2) are expressed as follows:^[^ ^69^ ^]^

(1) $$\tau_{dy}\propto H^{m}\left(m\approx 2\right)\;\;H\leq H_{critical}$$

(2) $$\tau_{dy}\propto H^{m}\left(m\approx 1.5\right)\;\;H\geq H_{critical}$$

Here, m is defined as the power law exponent, which can be reflected by solving the relationship between dynamic yield strength and magnetic field intensity, and is related to the properties and concentration of the particles.

The dynamic yield strength analysis of MRFs‐15/85 is shown in Figure 6d. It can be observed that the values of m at low and high magnetic fields are 2 and 1.2, respectively, deviating from the theoretical values (2 and 1.5). The main reason for this deviation is the lower saturation magnetization of FeNi particles, which limits their effectiveness at high magnetic fields. However, the m values for MRFs‐0/100 are only 1.6 and 1.4 (refer to Figure S11d, Supporting Information), respectively, also lower than the theoretical values, primarily due to the lower particle concentration specified in this study. Notably, the m value for MRFs‐15/85 with low magnetic field conditions is significantly higher than that for MRFs‐0/100, indicating that this bidisperse system exhibits a more pronounced response to magnetic fields at low levels, thereby forming more stable chain‐like structures. Compared with our previous work,^[^ ^45^ ^]^ the degree of change of the FeNi bidisperse system MRFs under the medium and low magnetic field is increased from 1.8 to 2, indicating a higher adjustable range.

The time response capability and reversibility of MRFs are crucial for their stable and continuous operation in devices. Figure 6e depicts the behavior of shear stress changes corresponding to instantaneous variations in magnetic field intensity under constant shear rate (10 s^−1^) for MRFs‐15/85. It can be observed that the shear stress response of MRFs‐15/85 to magnetic fields is instantaneous and independent of the magnitude of the magnetic field. Importantly, regardless of the extent of magnetic field gradient changes, the performance of MRFs‐15/85 can recover almost entirely to its initial state. This indicates that the bidisperse particle system exhibits excellent instantaneous and stable response capabilities comparable to monodisperse systems, and own uniquely superior redispersibility.^[^ ^70^ ^]^ Flow curves under zero field conditions before and after applying the highest magnetic field (sustained for 1 min), which reflect details of the reversibility of MRFs‐15/85, as shown in Figure 6f. The results demonstrate that the average discrepancy in flow curves before and after applying the highest magnetic field is less than 1%. Compared to MRFs‐0/100 (1.3%, refer to Figure S11f, Supporting Information), the bidisperse system exhibits better reversible usability. This is attributed to the lower coercivity and remanence of FeNi particles, which effectively inhibit large‐scale and irreversible aggregation among the primary particles (CIPs) responsible for producing MR effects. Additionally, submicron particles, unlike nanoparticles, are less prone to self‐aggregation, thereby avoiding phenomena such as adsorption and accelerated CIPs aggregation. Compared to our previous work,^[^ ^45^ ^]^ the difference in the performance of this FeNi bidisperse system MRFs after dispersion was reduced from 1.5% to 1.0%, indicating a more remarkable reversible usability. Further, we tested the properties of the settled MRFs‐15/85 after redispersion, and found that there was no significant change in MR effect (refer to Figure S12, Supporting Information).

## 3 Conclusion

This study prepared submicron FeNi particles using the DC arc plasma method, with an average particle size of 0.42±0.16 µm, saturation magnetization of 167.2 emu g^−1^, remanent magnetization of 0.8 emu g^−1^, and coercivity of 16.5 Oe. Furthermore, a bidisperse MRFs was developed by doping these submicron FeNi particles with CIPs, exhibiting high shear yield strength at low magnetic fields. Comparative analysis based on shear yield strength, sedimentation stability, and zero‐field viscosity index model concluded that MRFs‐15/85 demonstrated superior comprehensive performance. Specifically, the yield strength reached 19.1 kPa at 230 mT magnetic field (a 38.4% improvement over pure CIPs based MRFs), sedimentation stability was 65.1% over 10 days, and zero‐field viscosity was 1.06 Pa s at 100 s^−1^. The contribution mechanisms of parameters such as chain‐like structure, magnetic permeability, magnetic flux density, and friction coefficient to shear yield strength were elucidated. Results indicate that the interspaces filling effect of high magnetic permeability submicron FeNi particles increased the compactness of chain‐like structures, enhancing both their shear strength resistance and magnetic field permeability at low fields, thereby collectively boosting shear yield strength. Introduction of submicron particles reduced overall particle density, enhancing sedimentation stability of the MRFs without significantly increasing zero‐field viscosity. The low remanent magnetization characteristics of FeNi particles also imparted excellent redispersibility and reversibility to the bidisperse MRFs. Additionally, detailed characterization and analysis of flow curves, static shear yield stress, magnetic field responsiveness, and reversibility of MRFs‐15/85 confirmed its practical application capabilities. This study's straightforward preparation method for MRFs with high comprehensive performance lays a foundation for industrial development of predominant performance magnetorheological fluids.

## 4 Experimental Section

### Materials

The FeNi alloy (99.6% purity, Fe‐Ni ratio 1:1) used for synthesizing submicron FeNi particles was procured in bulk from the X‐power Material Technology (Dalian) CO. Ltd., 1J50 alloy, requiring no further purification before use. The CN series CIPs were purchased from BASF (Shanghai, China), with an average diameter of ≈2.70 µm and Fe content exceeding 99.5%. The dimethyl silicone oil was obtained from Shanghai Aladdin Bio‐Chem Technology Co., Ltd., with a dynamic viscosity of 200.0 mPa s and density of 0.96 kg m^−3^.

### Preparation of Particles and MRFs

**Figure 7:**
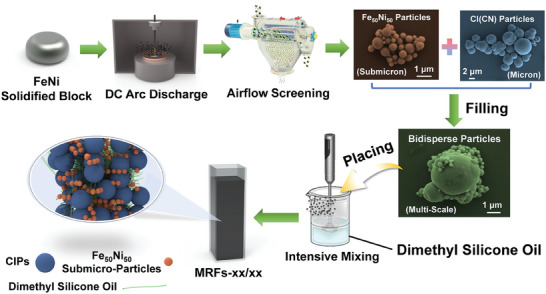
Schematic diagram for the fabrication process of the Submicron FeNi particles and CIPs‐FeNi bidisperse MRFs.

**Figure** **7** depicts the preparation process of the submicron FeNi particles and bidisperse MRFs as a whole. Prior to the synthesis process, the DC arc plasma apparatus must be evacuated to a vacuum pressure of 0.1 MPa, followed by the introduction of Ar to establish an absolute vacuum environment at 0.08 MPa. Within this controlled environment, the FeNi alloy ingot was subjected to complete melting, resulting in a block material that conforms precisely to the geometry of the molten pool. Subsequent to melting, the material was allowed to cool for a duration of 30 min. Thereafter, a precise quantity of H_2_ was introduced to achieve an absolute vacuum pressure of 0.06 MPa, with a stoichiometric volume ratio of hydrogen to nitrogen maintained at 1:1 within the apparatus. The synthesis of submicron FeNi particles was initiated by activating the plasma arc gun and fine‐tuning the electrical current to a set parameter of 200 A. This process continues until no further particle formation was observed, at which point the plasma arc gun was deactivated. Subsequently, the apparatus was subjected to a cooling regimen using cooling water for ≈30 min to ensure uniform temperature reduction. To mitigate the risk of explosive reactions, a controlled amount of air was introduced to passivate the system for a period of 6 h. Ultimately, the high‐purity submicron FeNi particles were isolated through a combination of air jet separation and sieving techniques, thereby completing the preparation process. A more detailed preparation process could also be found in the earlier work.^[^ ^71^ ^]^

For the preparation of bidisperse MRFs, submicron FeNi particles and CIPs were pre‐mixed in varying proportions and added together to a beaker containing dimethyl silicone oil, maintaining a particle content of 65 wt.%. The mixture was thoroughly stirred at room temperature to achieve uniform suspension, thereby preparing a series of MRFs for experimental research. The final distribution of particles in the carrier liquid could be inferred from the schematic diagram provided. Given the study's focus on particle systems for innovation and research purposes, the system was deliberately simplified without the introduction of additional additives. All prepared MRFs were immediately used for experimental testing to ensure consistent experimental conditions without variation due to storage time.

### Characterization of Particles and MRFs

The morphology and compositional analysis of submicron FeNi particles and CIPs were thoroughly investigated using a JSM‐7900F ultra‐high resolution field‐emission scanning electron microscope (FE‐SEM) coupled with an energy dispersive spectrometer (EDS). The phase structure of submicron FeNi nanoparticles and CIPs was extensively characterized using a Bruker D8 Advance X‐ray diffractometer (Cu target and 2.2 kW ceramic X‐ray tube) produced by Bruker Corporation. The magnetic properties of submicron FeNi particles and CIPs were evaluated over a range of ‐15 000 to 15 000 Oe using a Lakesore‐7400s vibrating sample magnetometer (VSM) at 40 °C.

The rheological performance of MRFs containing varying concentrations of bidisperse particles were investigated at 40 °C using an Anton Paar MCR‐301 rheometer (parallel plate system with PP‐20 geometry, 1 mm test gap distance, and magnetic field intensity ranging from 0 to 545 mT). The zero‐field viscosity was separately measured using a coaxial cylinder test system (CC27). It was noteworthy that a fixed volume of 0.75 mL was used for all MRF samples to avoid volume changes affecting the MR performance. Sedimentation stability of different bidisperse MRFs was evaluated by calculating the percentage of sedimentation height to the initial MRFs height under conditions perpendicular to the liquid surface.

### Statistical Analysis

All data and graphical representations were conducted using Origin 2022 software. Except for the particle size distribution statistics, shear yield strength, and power‐law exponent, all other data are unprocessed raw data. The particle size distribution graph was determined using ImageJ and Origin 2022 in conjunction, and a statistical significance test was performed, with the average particle size expressed in the form of mean ± SD. The yield strength and power‐law exponent were calculated based on the corresponding raw values using established formulas in Origin 2022. All data related to performance measurements were based on at least four samples of the same composition, and no significant differences were observed.

## Conflict of Interest

The authors declare no conflict of interest.

## Supporting information



Supporting Information

## Data Availability

The data that support the findings of this study are available from the corresponding author upon reasonable request.
