# Supplementary material for: Significant Improvement in Magnetorheological Performance by Controlling Micron Interspaces with High Permeability Submicron Particles
Source: Adv Sci (Weinh). 2024 Oct 8;11(44):2407765. doi: 10.1002/advs.202407765 (PMC11600251; doi:10.1002/advs.202407765)
Supplement: Supplementary file 1 — Supporting Information [file ADVS-11-2407765-s001.docx]

Supporting Information

**Significant Improvement in Magnetorheological Performance by Controlling Micron Interspaces with High Permeability Submicron Particles**

Tianxiang Du^a^, Ning Ma^b^, Zenghui Zhao^a^, Yitong Liu^a^, Xufeng Dong^a,*^, Hao Huang^a,*^

T. Du, N. Ma, Z. Zhao, Y. Liu, Prof. X. Dong, Prof. H. Huang

^1^School of Materials Science and Engineering, Dalian University of Technology,

Dalian, 116024, P. R. China.

^2^State Key Laboratory of Coastal and Offshore Engineering, School of Civil Engineering, Dalian University of Technology, Dalian, China.

E-mail: dongxf@dlut.edu.cn; huanghao@dlut.edu.


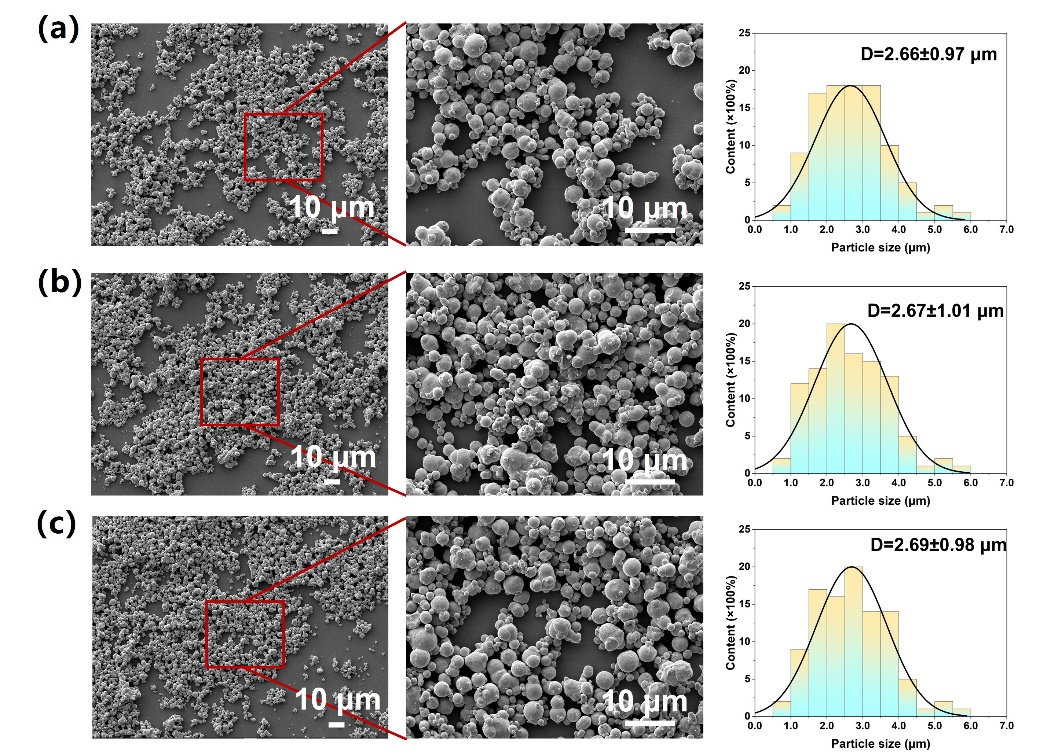


**Figure S1.** SEM images and particle size distribution statistics of different batches of CIPs.


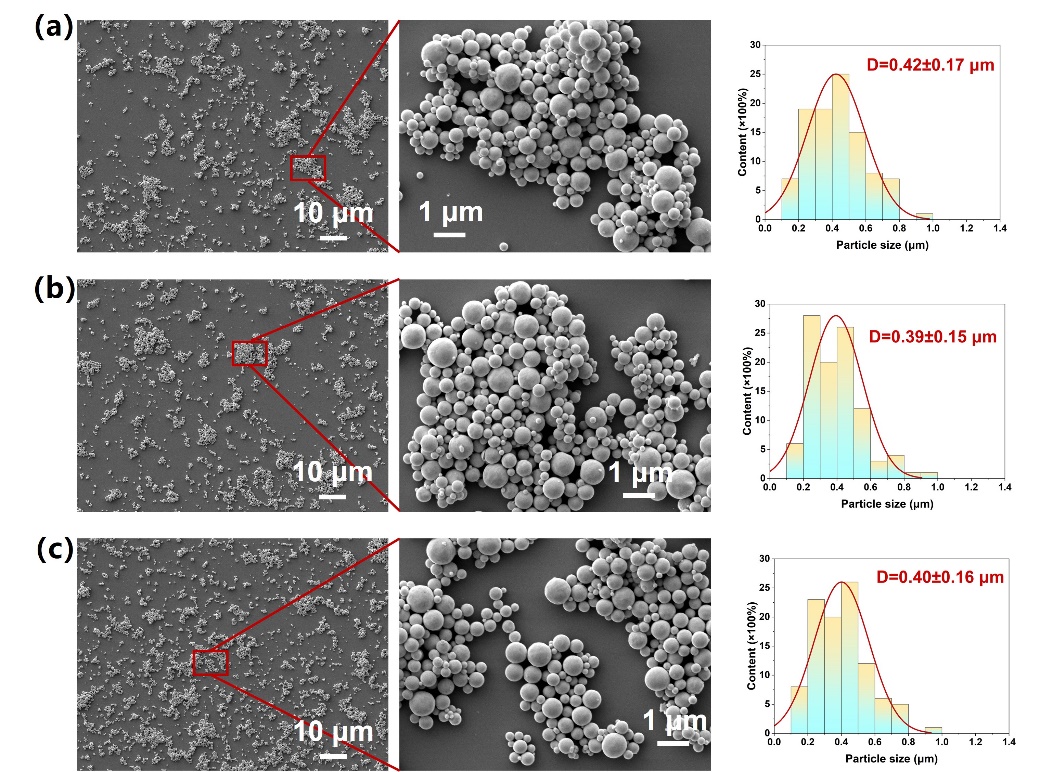


**Figure S2.** SEM images and particle size distribution statistics of different batches of submicron FeNi.


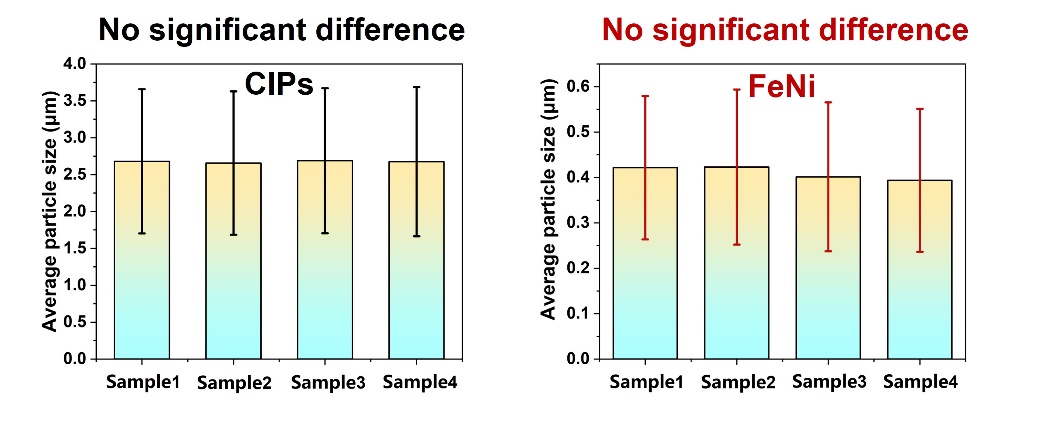


**Figure S3.** Statistical significance of the size distribution of CIPs and submicron FeNi.

**Table S1.** Comparison of properties of different particles applied to MRFs

| **Preparation method** | **Particle type** | ***M_s_* (emu/g)** | ***H_c_* (Oe)** | ***M_r_* (emu/g)** | **Particle size (nm)** | **Reference** |  |
| --- | --- | --- | --- | --- | --- | --- | --- |
| DC arc method | Fe_50_Ni_50_ | 167.2 | 16.5 | 0.8 | 420±160 | This work |  |
| Chemical method | Fe_50_Ni_50_ | 109 | 169 | ≈ 9 | 50 ~ 100 | [1] |  |
|  |  |  |  |  |  |  |  |
| Chemical method | Fe_71_Ni_29_ | 78 | 174 | ≈ 9 | 20 ~ 100 | [1] |  |
| DC arc method +anneal dispose | Fe_50_Co_50_ | 208.0 | 58 | 5.8 | 50 ~ 470 | [2] |  |
| DC arc method | Fe | 146 | 500 | 29.5 | 30 ~ 200 | [3] |  |
| Chemical method | Co_50_Ni_50_ | 140.1 | ≈ 240 | ≈ 19 | ≈ 1000 | [4] |  |
| Chemical method | Co_90_Ni_10_ | 119.5 | 142 | 21 | ≈ 450 | [5] |  |
| Chemical method | Fe_3_O_4_ | 82 | 35 | 2.3 | ≈ 450 | [6] |  |


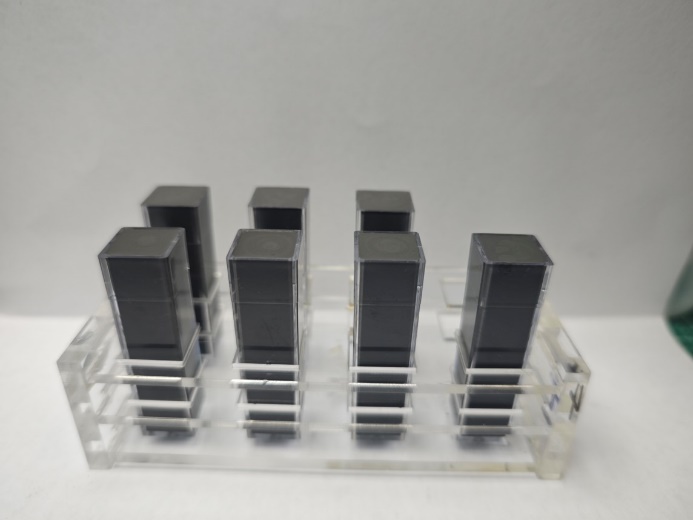


**Figure S4.** Placement of different kinds of MRFs during natural sedimentation


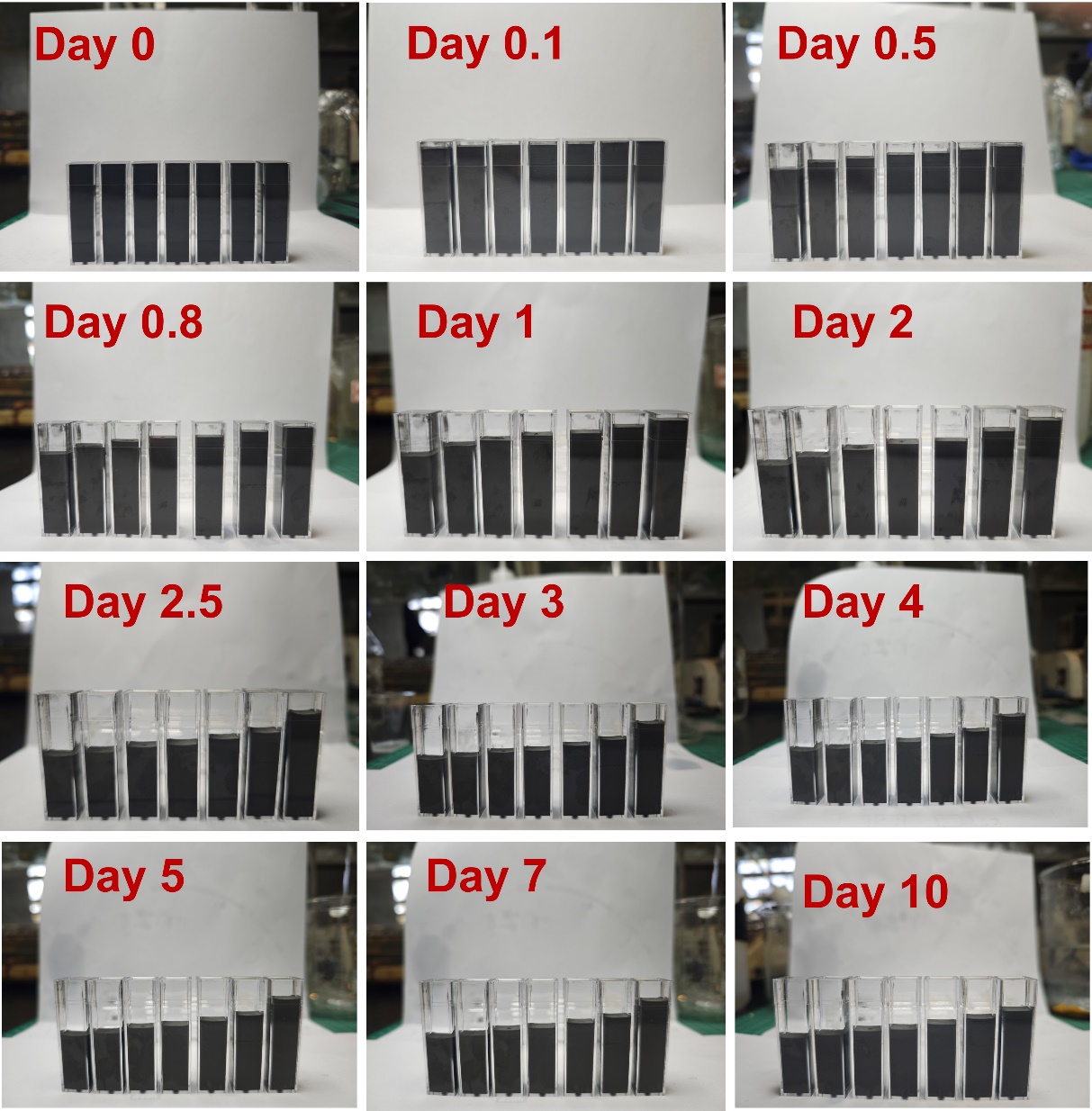


**Figure S5.** Sedimentation photos of different kinds of MRFs at different time points

**Table S2.** Comparison of properties of other silicon-based bidisperse MRFs

| **Particle Type** | **Mass Fraction (wt.%)** | **Shear Yield Strength（kPa）** | **Zero-Field Viscosity (Pa, 100 s^-1^)** | **Sedimentation Stability** | **References** |
| --- | --- | --- | --- | --- | --- |
| CIPs with 15 wt.% FeNi | 65 | 19.1 (230 mT) | 1.06 | 72.5 % (48 h) | This work |
| CIPs with 10 wt.% Nano FeCo | 65 | 14.2 (230 mT) | 1.25 | 91.1% (48 h) | [7] |
| CIPs with 25 wt.% Nano Fe | 40 | 12.5 (230 mT) | 0.6 | 56.1% (60 h) | [8] |
| CIPs with 4 wt.% nanoFe_3_O_4_ | 65 | ≈ 15 (250 mT) | ≈ 2.8 | 80% (60 h) | [9] |
| CIPs+0.1 wt.%  Nano Fe_3_O_4_/sepiolite | 50 | ≈ 5 (214 mT) | ≈ 4 | 60% (40 h) | [10] |
| CIPs+0.1 wt.% PANI | 52 | 3.88 (214 mT) | 1.2 | 68% (20 h) | [11] |
| CIPs+0.1 wt.%  Nano Fe_3_O_4_@mSiO_2_ | 50 | ≈ 4 (214 mT) | ≈ 4 | 50% (40 h) | [12] |


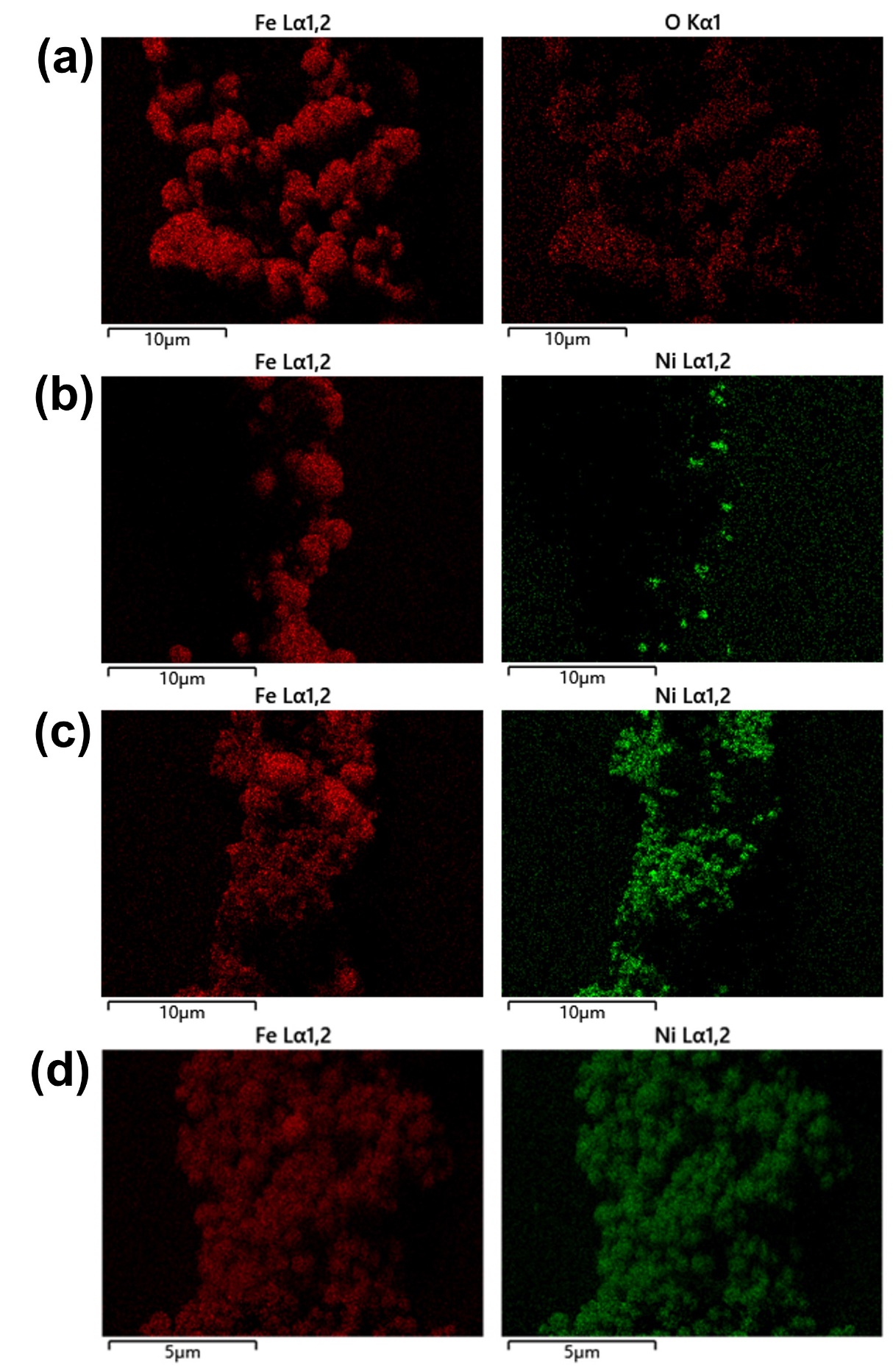


**Figure S6.** The EDS images of CIPs-FeNi bidisperse MRFs chain-like structures with different particles proportions. The images of MRFs-0/100 (a), MRFs-15/85 (b), MRFs-30/70 (c) and MRFs-100/0 (d).


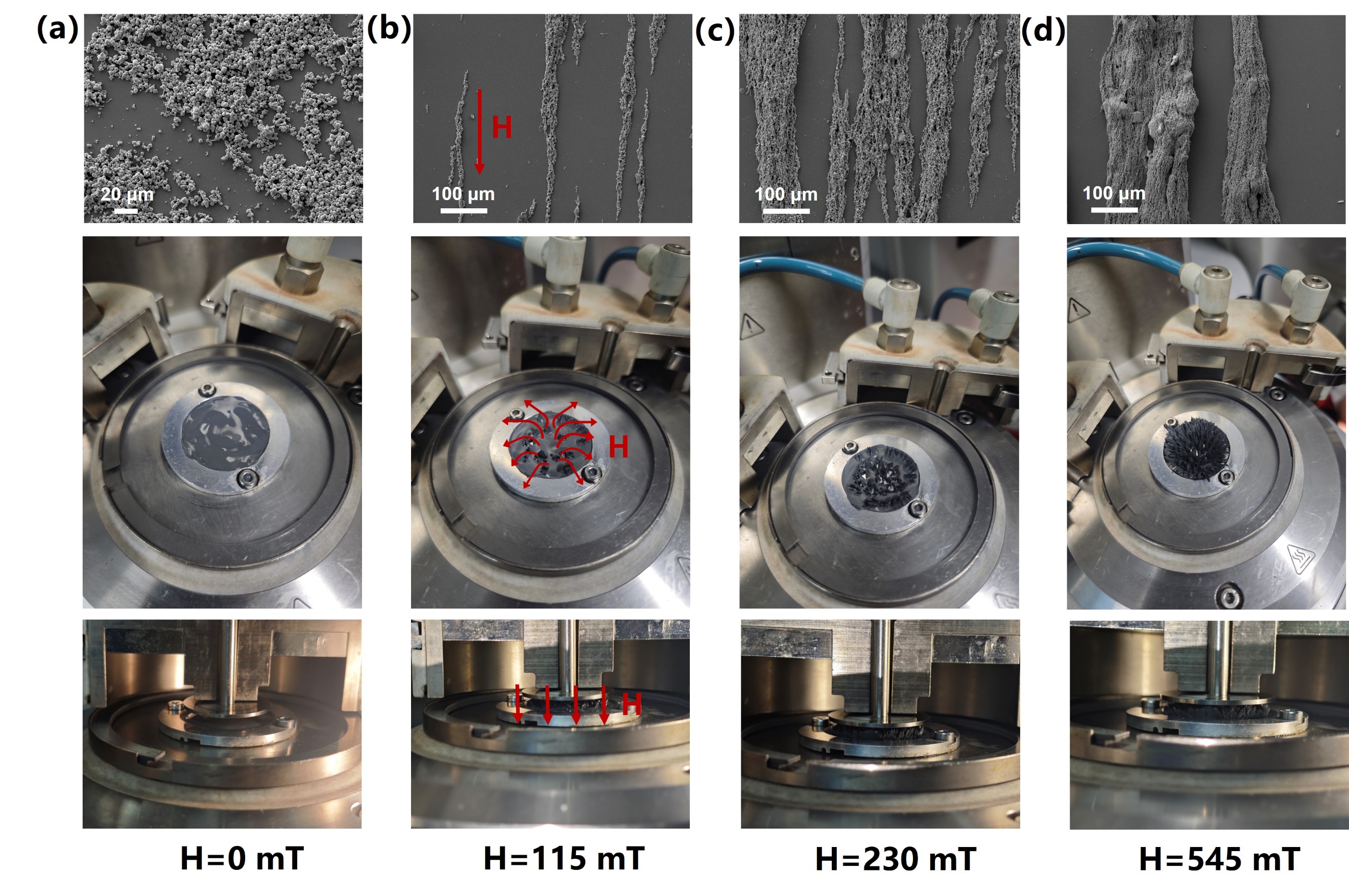


**Figure S7.** The Microscopic and macroscopic behavior characterization of MRFs-15/85 under different magnetic fields strength. 0 mT (a), 115 mT (b), 230 mT (c) and 545 mT (d).


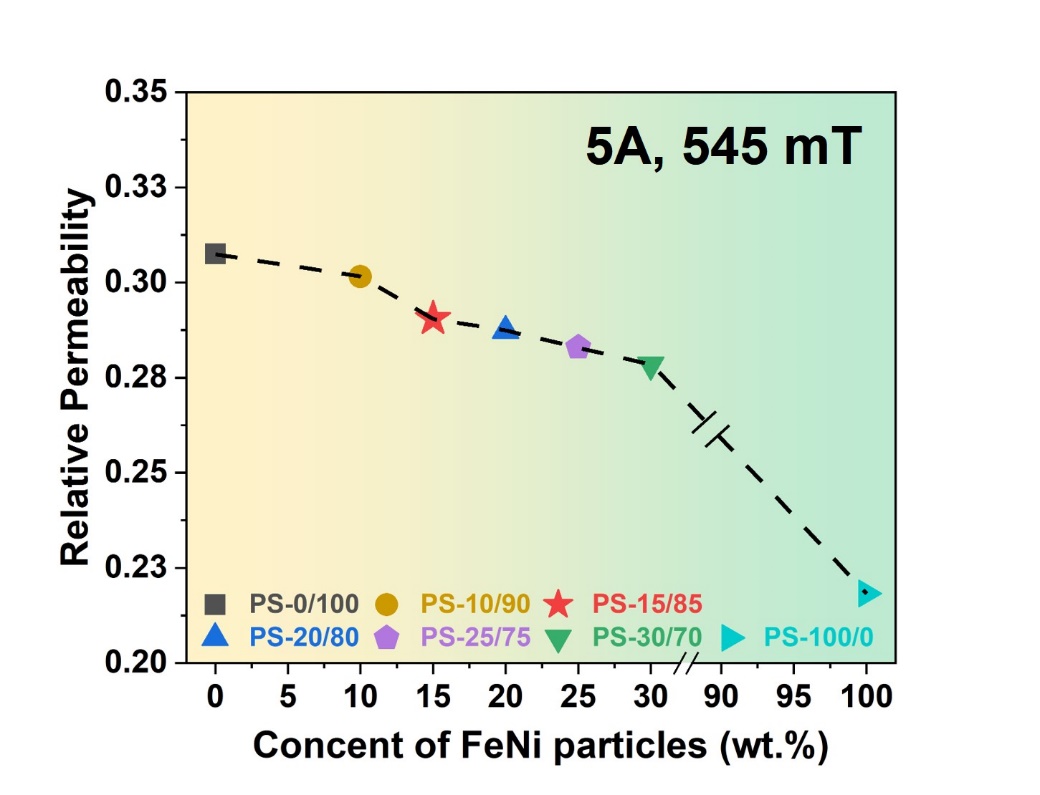


**Figure S8.** The relative permeability value (from VSM) at 545 mT magnetic field.


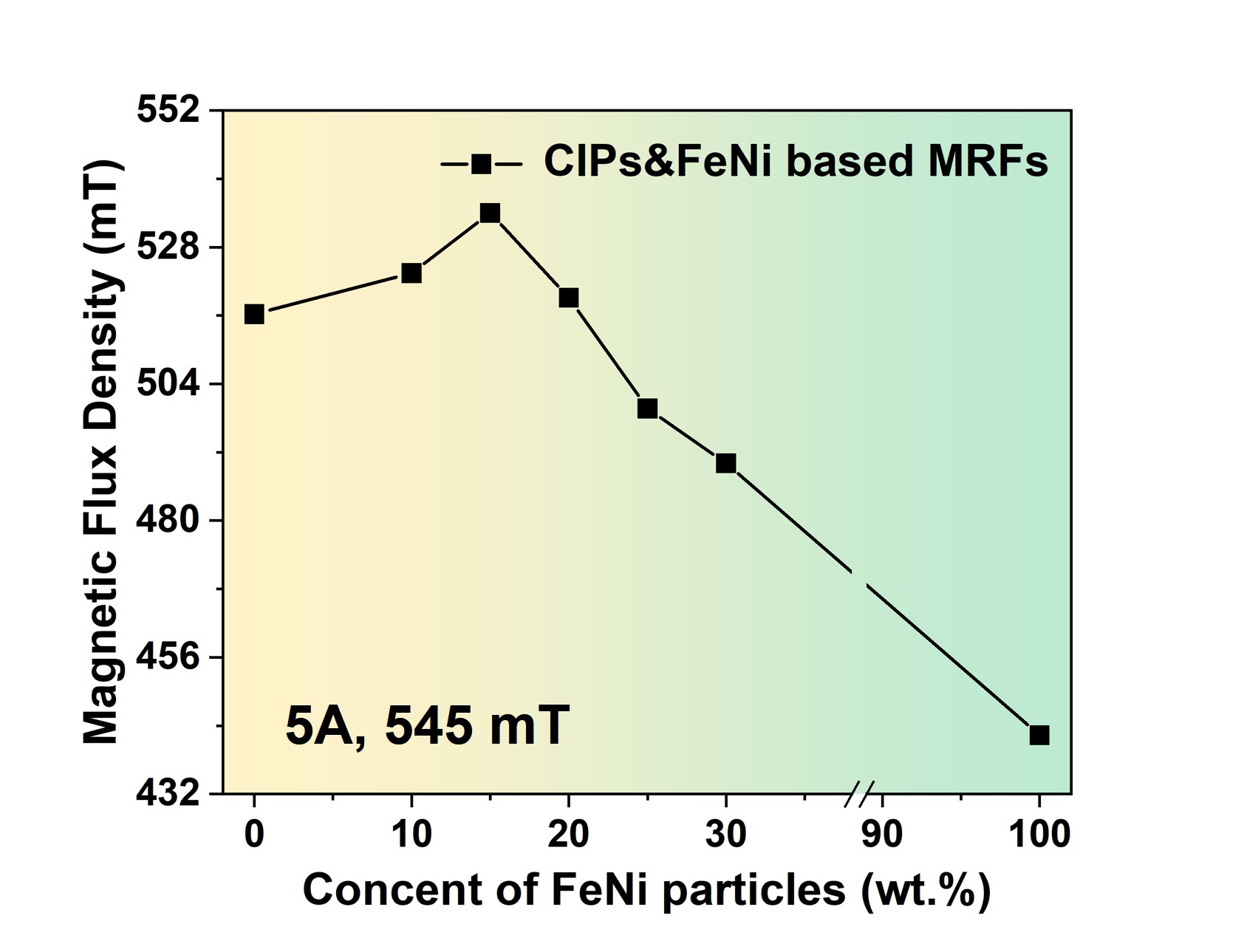


**Figure S9.** The Magnetic Flux Density of different particles proportions MRFs in 545 mT magnetic field


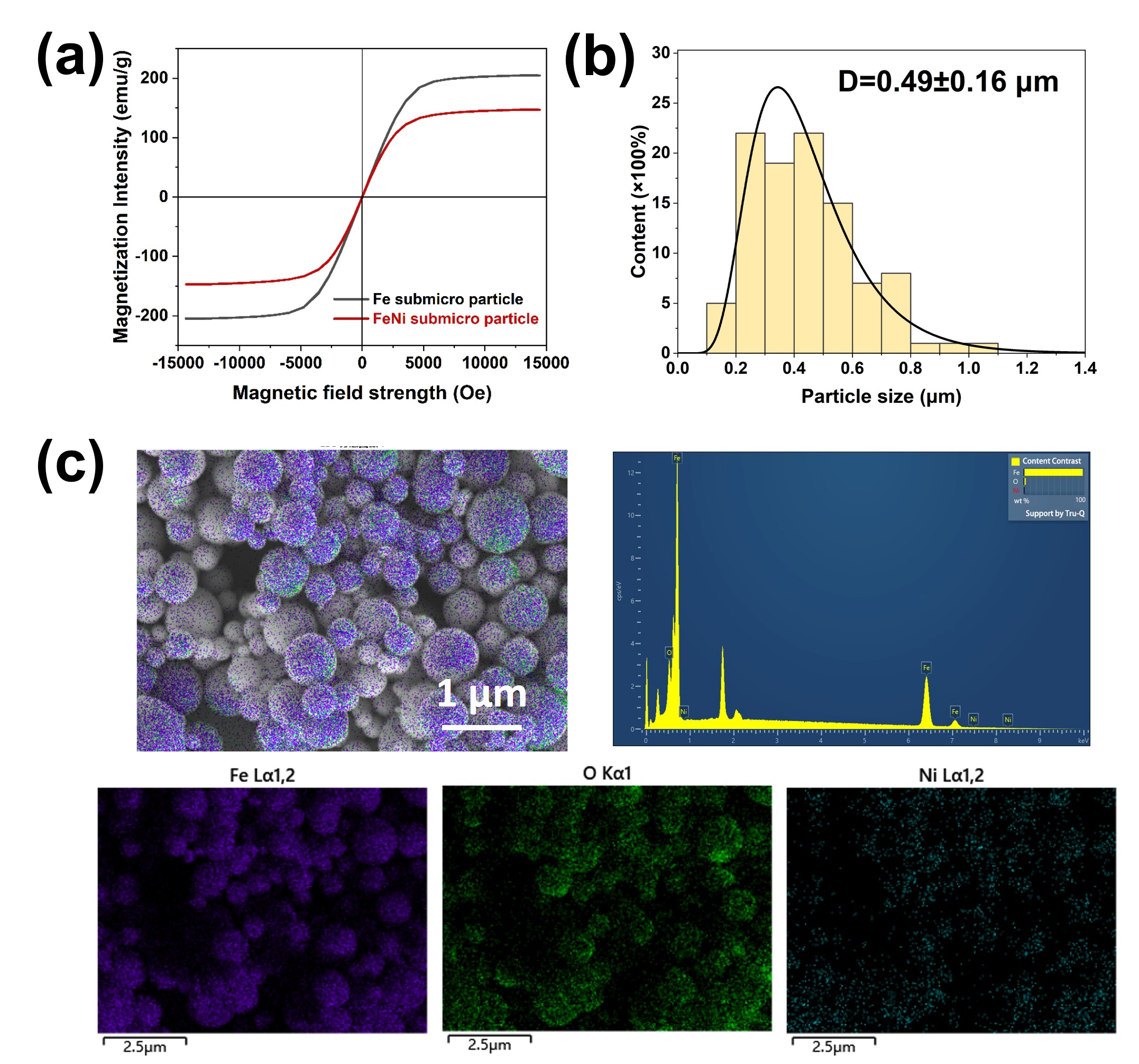


**Figure S10.** The VSM comparison of submicron Fe and FeNi particles (a), the PSD (b) and EDS (c) analysis of submicron Fe particles


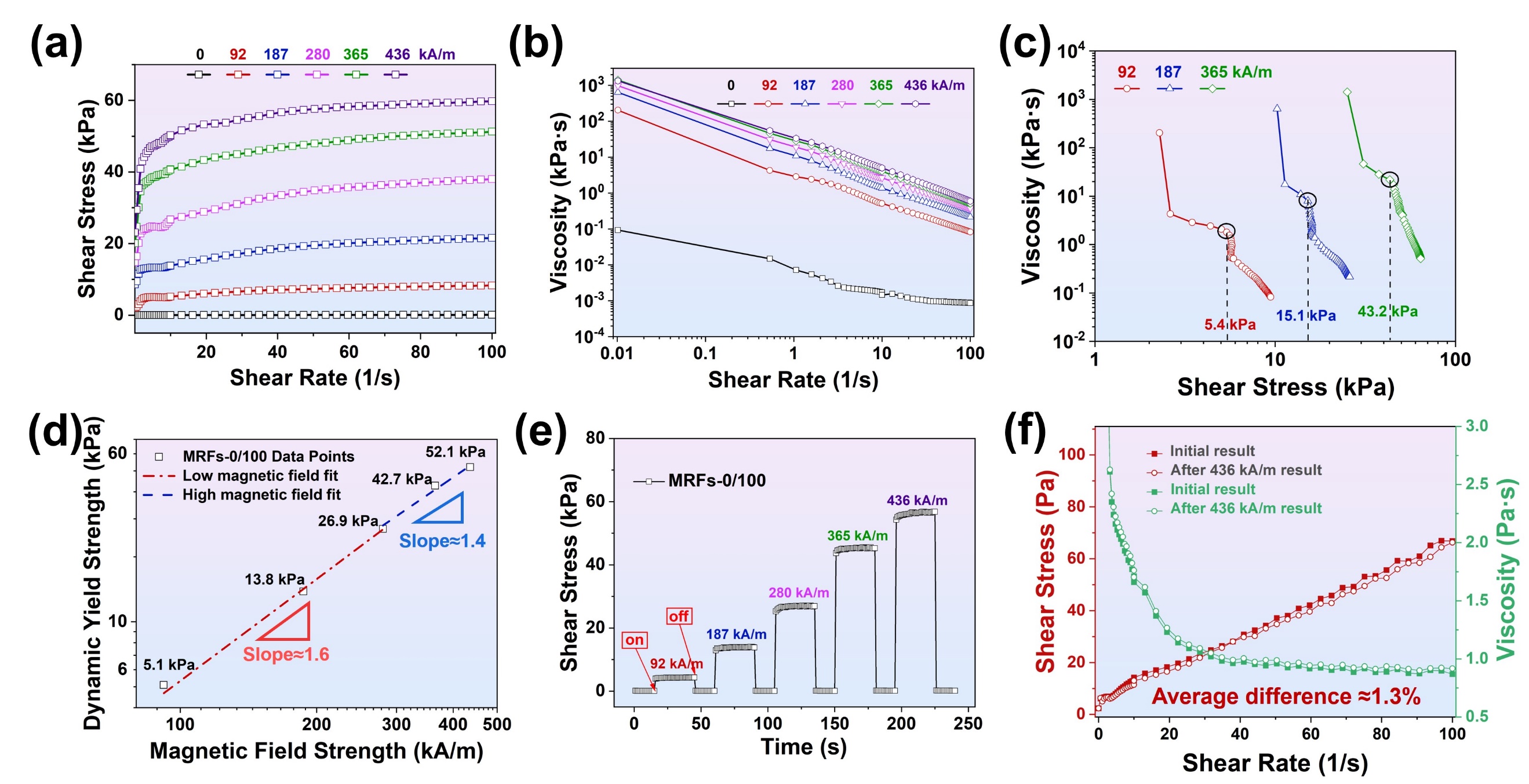


**Figure S11.** The other MR performance curves of MRFs-0/100 at steady state mode. The curve of shear stress vs. shear rate (a), viscosity vs. shear rate(b), static yield strength (c), dynamic yield strength (d), time responsiveness images (e) and reversibility (f) of MRFs-0/100.


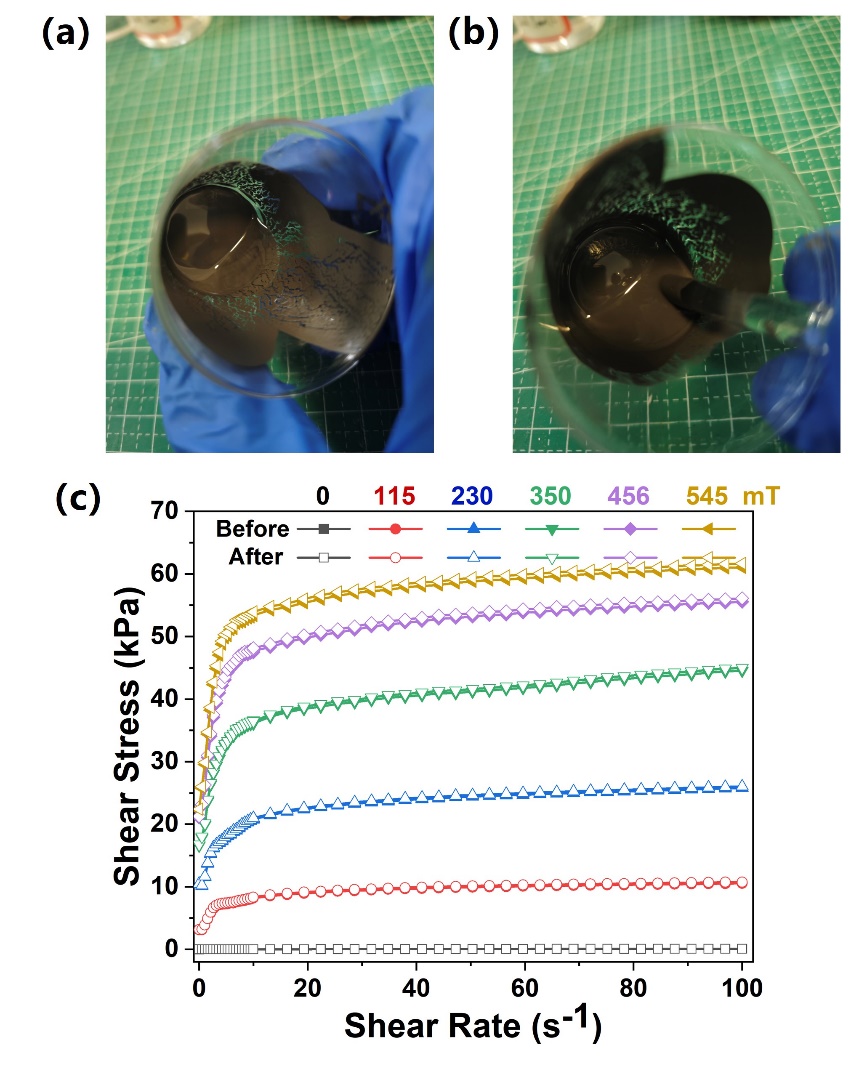


**Figure S12.** Performance test of settled MRF-15/85 samples after redispersing. The settled MRF-15/85 (a), the redispersed MRF-15/85 (b), the performance comparison of initial MRF-15/85 and redispersed MRF-15/85.

**Reference**

[1] A. Wu, J. Gao, X. Chen, X. Yang, H. Yang, *J ALLOY COMPD* **2014**, *583*, 55.

[2] T. Du, P. Zhao, Y. Tong, N. Ma, H. Huang, X. Dong, *NANOTECHNOLOGY* **2023**, *34*, 115701.

[3] W. Zhu, X. Dong, H. Huang, M. Qi, *J MAGN MAGN MATER* **2019**, *491*, 165556.

[4] W. L. Zhang, F. Qu, Y. Tian, H. J. Choi, L. Deng, J. Liu, J. Tian, H. Liu, *ADV MATER INTERFACES* **2018**, *5*, 1800164.

[5] I. Arief, P. K. Mukhopadhyay, *J MAGN MAGN MATER* **2016**, *397*, 57.

[6] A. V. Anupama, V. Kumaran, B. Sahoo, *J IND ENG CHEM* **2018**, *67*, 347.

[7] T. Du, P. Zhao, Y. Liu, N. Ma, X. Dong, H. Huang, *ADV FUNCT MATER* **2024**, *34*, 2311254.

[8] W. Zhu, X. Dong, H. Huang, M. Qi, *J INTEL MAT SYST STR* **2020**, *32*, 1271.

[9] X. Liu, L. Wang, H. Lu, D. Wang, Q. Chen, Z. Wang, *MATER MANUF PROCESS* **2015**, *30*, 204.

[10] Y. Z. Dong, W. J. Han, H. J. Choi, *J IND ENG CHEM* **2021**, *93*, 210.

[11] F. F. Fang, Y. D. Liu, H. J. Choi, Y. Seo, *ACS APPL MATER INTER* **2011**, *3*, 3487.

[12] J. H. W., J. C. H., *IEEE T MAGN* **2018**, *54*, 1.
